# Effects of refractive errors on visual evoked magnetic fields

**DOI:** 10.1186/s12886-015-0152-6

**Published:** 2015-11-09

**Authors:** Masaya Suzuki, Mizuki Nagae, Yuko Nagata, Naoya Kumagai, Koji Inui, Ryusuke Kakigi

**Affiliations:** Department of Integrative Physiology, National Institute for Physiological Sciences, Okazaki, 444-8585 Japan; R&D Department, Tokai Optical Co., Ltd, Okazaki, 444-2192 Japan

**Keywords:** Magnetoencephalography, Primary visual cortex, Refractive error, Visual evoked magnetic field

## Abstract

**Background:**

The latency and amplitude of visual evoked cortical responses are known to be affected by refractive states, suggesting that they may be used as an objective index of refractive errors. In order to establish an easy and reliable method for this purpose, we herein examined the effects of refractive errors on visual evoked magnetic fields (VEFs).

**Methods:**

Binocular VEFs following the presentation of a simple grating of 0.16 cd/m^2^ in the lower visual field were recorded in 12 healthy volunteers and compared among four refractive states: 0D, +1D, +2D, and +4D, by using plus lenses.

**Results:**

The low-luminance visual stimulus evoked a main MEG response at approximately 120 ms (M100) that reversed its polarity between the upper and lower visual field stimulations and originated from the occipital midline area. When refractive errors were induced by plus lenses, the latency of M100 increased, while its amplitude decreased with an increase in power of the lens. Differences from the control condition (+0D) were significant for all three lenses examined. The results of dipole analyses showed that evoked fields for the control (+0D) condition were explainable by one dipole in the primary visual cortex (V1), while other sources, presumably in V3 or V6, slightly contributed to shape M100 for the +2D or +4D condition.

**Conclusions:**

The present results showed that the latency and amplitude of M100 are both useful indicators for assessing refractive states. The contribution of neural sources other than V1 to M100 was modest under the 0D and +1D conditions. By considering the nature of the activity of M100 including its high sensitivity to a spatial frequency and lower visual field dominance, a simple low-luminance grating stimulus at an optimal spatial frequency in the lower visual field appears appropriate for obtaining data on high S/N ratios and reducing the load on subjects.

## Background

Visual evoked potentials (VEPs) or magnetic fields (VEFs) are useful for assessing the visual system [1, 2]. However, VEPs are affected by non-pathological factors such as age, sex, and refractive states as well as visual stimulus parameters such as spatial frequency, contrast, and luminance. Of these, the refractive error is important because the latency and amplitude of VEPs are both markedly affected by a blur in the retina [3–5], particularly when the spatial frequency of the stimulus is high [4, 6–8]. Therefore, correction of refractive errors is necessary for avoiding false positive results in clinical tests. On the other hand, this suggests that the VEP amplitude and latency are applicable to objective assessments of refractive errors [9, 10]. We consider VEPs with appropriate controls applicable as a tool for developing a better lens or prescribing a tailor-made lens for individuals.

In order to establish an easy and reliable method to objectively assess the eyes of a subject, we conducted a series of experiments using VEPs and VEFs. We herein described one of these experiments using transient VEFs. Although two fMRI studies previously demonstrated that induced myopia reduced visual evoked cortical activity in Brodmann’a areas 17 and 18 [11] or in V1 and V2 [12], there is currently no electrophysiological study that describes neural origins of refraction-sensitive activity. We employed a low-luminance simple grating in the present study instead of a pattern reversal stimulus in order to reduce the overall luminance of the stimulus by considering subject discomfort. No significant differences were observed in the effects of checks and gratings of high spatial frequencies on VEPs [13].

## Methods

Twelve (four females and eight males) healthy right-handed volunteers, aged 24–47 years (33.0 ± 6.7), with normal corrected visual acuity (20/20) and without neurological and ophthalmic disorders were enrolled. The study was approved in advance by the Ethics Committee of the National Institute for Physiological Sciences, Okazaki, Japan, and written consent was obtained from all subjects.

### Stimulus

**Fig. 1:**
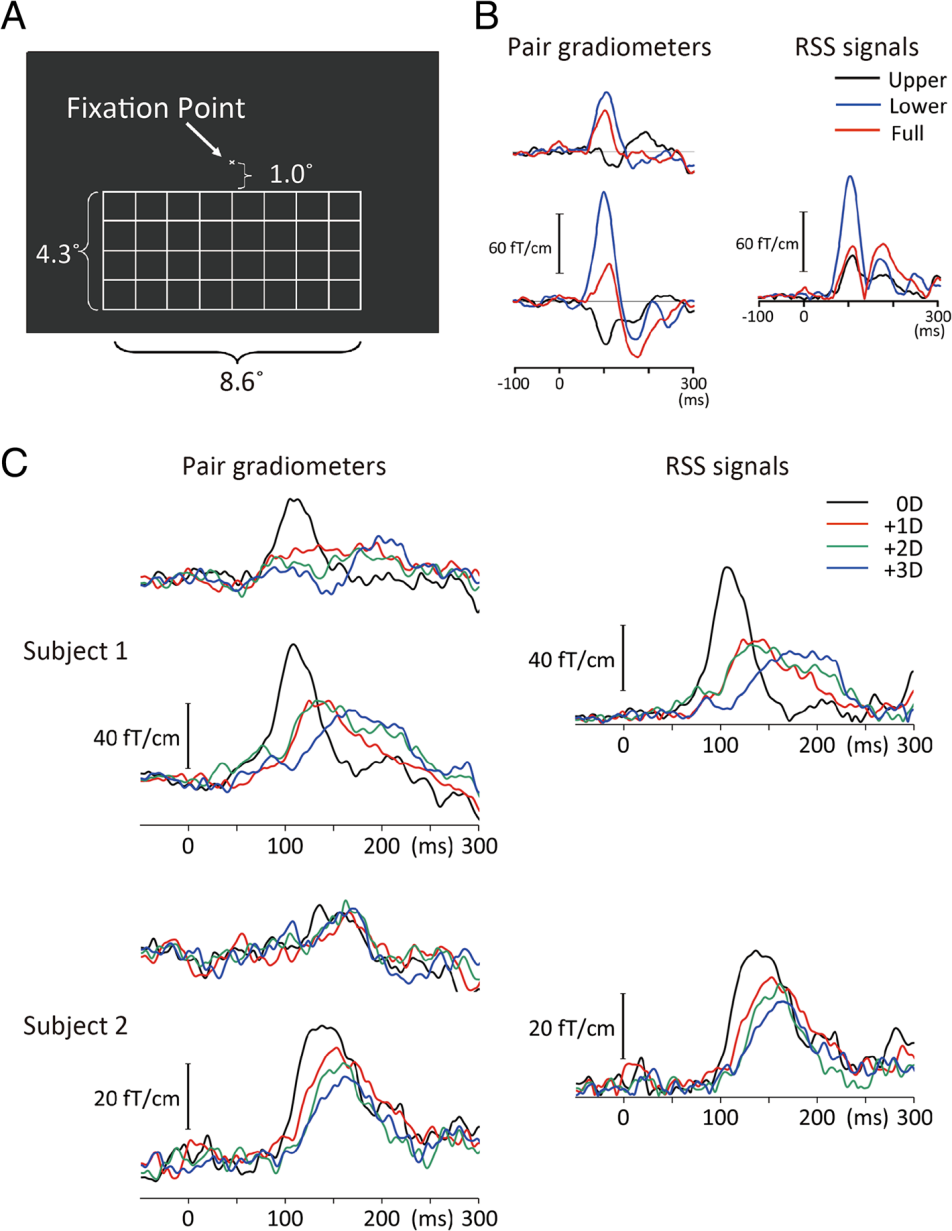
Magnetic responses in representative subjects. **a** Stimulus. **b** magnetic waveforms in pair gradiometers with the greatest response, and root mean square (RSS) signals of pair gradiometers in a representative subject. Responses to the upper, lower, and full-field stimulations are shown. Note that responses to the lower and upper visual field stimulations were opposite in polarity and that the response to the full-field stimulation was approximately the sum of the two. **c** RSS waveforms for four refractive states obtained from selected pair gradiometers in two subjects

The visual stimulus was presented on a screen by a digital light processing projector placed outside of a shielded room (Mirage 2000, Christie Digital System Inc., Kitcherner, Canada). The refresh rate of the projector was 60 Hz. We used a simple grating (Fig. 1a). Subjects were seated in front of the screen at a viewing distance of 2 m. The viewing angle of the grating stimulus was 4.3° (vertical) X 8.6° (horizontal) when it was presented in the lower or upper visual field. The width of the line was 0.06°. The luminance of lines was 0.16 cd/m^2^. The stimulus was 250 ms in duration and was presented every 500 ms. In Experiment 1, VEFs following upper, lower, and full-field stimulations were compared in nine subjects. Since the results obtained showed that the stimulus in the lower visual field elicited larger M100 than that in the upper visual field (Fig. 1b), the lower visual field was stimulated in 12 subjects in Experiment 2.

### Induction of refractive errors

Twenty-two out of the 24 eyes tested were myopic. Baseline recordings were initially made in the emmetropic state (+ 0D) by using lenses that the subjects typically wore. Myopia was induced by adding plus ophthalmic lenses of 1D, 2D, and 4D in a plastic trial frame for both eyes, and cortical responses were recorded for each condition in this order in all subjects.

### VEF recordings

This experiment was performed in a darkened magnetically shielded room. Binocular VEFs were recorded using a 306-channel whole-head type MEG system (Vector-view, ELEKTA Neuromag, Helsinki, Finland), which comprised 102 identical triple sensor elements. Each sensor element consisted of two orthogonal planar gradiometers and one magnetometer coupled to a multi-superconducting quantum interference device (SQUID), which provided 3 independent measurements of the magnetic fields. In this study, we analyzed MEG signals recorded from 204 planar-type gradiometers. These planar gradiometers were sufficiently powerful to detect the largest signal just over local cerebral sources. Signals were recorded with a bandpass of 1–200 Hz and digitized at 1004 Hz. An analysis was conducted from 100 ms before to 300 ms after the onset of each stimulus. The 100-ms pre-stimulus period was used as the baseline. Epochs with MEG signals larger than 2.7 pt/cm were rejected from averaging. Under each refractive condition, 100 artifact-free epochs were averaged.

### Analysis

We initially calculated vector sums from the longitudinal and latitudinal derivations of the responses recorded on planer-gradiometers at each of the 102 sensor locations. This was obtained by calculating the root sum square (RSS) of MEG signals of two gradiometers at the location of a sensor as described previously [14]. RSS waveforms were obtained for all 102 sensor locations and we selected one location around the occipital midline with maximal amplitude at a latency of 100–200 ms (major MEG component, M100). The peak latency and amplitude of M100 were then measured on the RSS waveform in each subject.

We subsequently performed a single-dipole analysis using the brain electric source analysis (BESA) software package (NeuroScan, Mclean, VA) as described elsewhere [15]. The locations of estimate dipoles were expressed in Talairach coordinates by using BrainVoyager (QX 1.4, Brain Innovation BV, Maastricht, The Netherlands). The latency and amplitude of M100 were measured in the source strength waveform obtained.

A one-way analysis of variance (ANOVA) was used for statistical comparisons of the latency, amplitude, and source location of M100 among the lens conditions tested. P values less than 0.05 were considered significant. Data are expressed as the mean ± standard deviation.

## Results

In Experiment 1, we compared VEFs in response to upper, lower, and full-field stimulations. Data from a representative subject are shown in Fig. 1b. The main component of VEFs at approximately 100 ms (M100) was opposite in polarity between the upper and lower visual field stimulations. The mean RSS values at the peak of M100 were 27.3 ± 10.6, 57.2 ± 30.5, and 39.1 ± 24.4 fT/cm for upper, lower, and full-field stimulations, respectively, with the differences observed being significant (F_2,14_ = 6.15, *p* = 0.012, partial η^2^ = 0.47). The peak amplitude was significantly greater for the lower than the upper visual field (*p* = 0.033). The visual field was not a significant factor for determining the latency of M100 (F = 0.19, *p* = 0.83). The effects of induced blurs were then examined by means of the lower visual field stimulation.

**Fig. 2:**
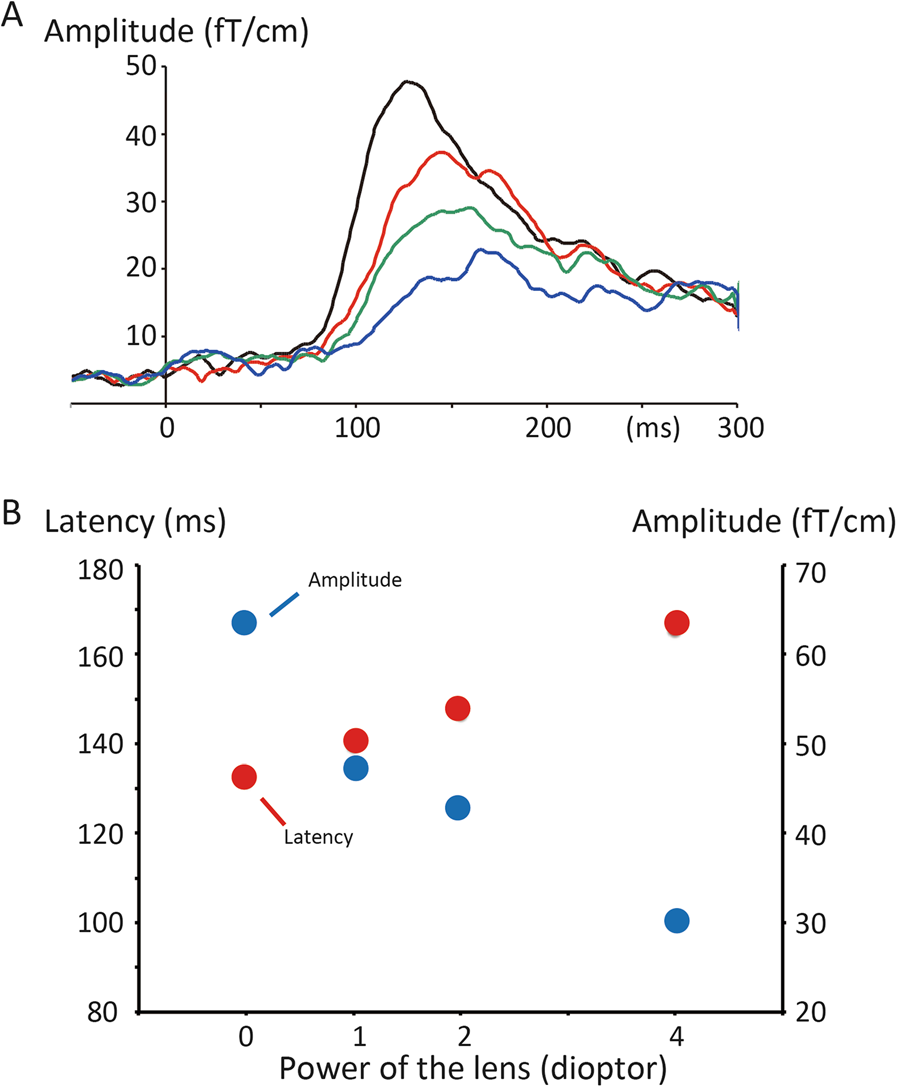
Effects of refractive errors on M100. **a** Grand-averaged RSS waveforms. **b** The peak latency and amplitude of M100 as a function of the power of lens

In Experiment 2, the visual stimulus evoked M100 at a sensor around the occipital midline in all subjects. Figure 1c shows the evoked magnetic fields and RSS waveform of M100 under each lens condition in two representative subjects. Grand-averaged RSS waveforms are shown in Fig. 2a. The amplitude gradually decreased and the latency became prolonged as the power of the lens increased (Fig. 2b). The results of one-way repeated measures ANOVAs showed that the refractive state was a significant factor for determining the latency (F_3,33_ = 22.36, *p* = 4.4 x 10^−8^, partial η^2^ = 0.67) and amplitude (F = 24.28, *p* = 1.8 x 10^−8^, partial η^2^ = 0.69) of M100. Post hoc tests showed that the latency (*p* = 0.001–0.021) and amplitude (*p* = 0.001–0.017) differed significantly between the 0D and three defocusing conditions. As shown in Fig. 2b, the function between the lens power (diopter) and latency or amplitude was almost linear.

**Fig. 3:**
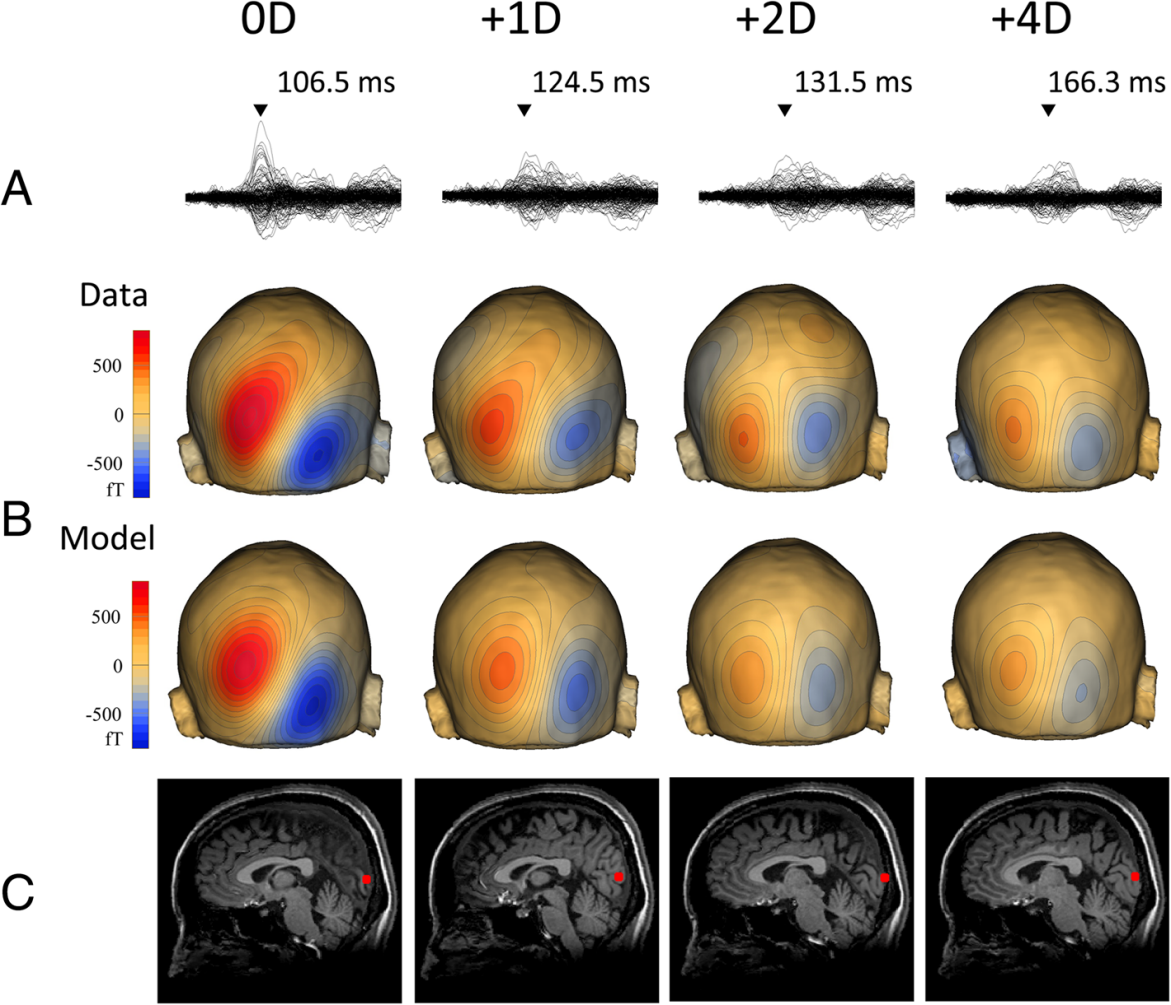
Single dipole analysis for M100. Data of a representative subject. **a** superimposed MEG waveforms recorded from all 204 channels. **b** Isocontour maps of measured and theoretical data at the peak of M100 for each lens condition. **c** Dipole locations superimposed on subject’s own MR images

The results of the single dipole analysis showed that a reliable dipole was obtained for the 0D and +1D conditions in ten subjects, for +2D in nine, and for +4D in six. Figure 3 shows an example of the dipole analysis. Similar to the RSS results, the source strength of M100 progressively decreased and its latency increased with stronger defocusing. When ANOVAs were applied to data from nine subjects in whom dipoles for the 0D, +1D, and +2D conditions were obtained, the latency (F_2,16_ = 9.22, *p* = 0.002, partial η^2^ = 0.54) and amplitude (F = 7.94, *p* = 0.004, partial η^2^ = 0.50) of M100 were significantly different among the three lens conditions. The mean location of the dipole in Talairach coordinates is listed in Table 1. A stronger lens caused a slightly superior dipole location. In order to confirm this statistically, we used data from the nine subjects. The results of one-way ANOVAs showed a significant difference among the three lens conditions for the z-axis (F_2,16_ = 7.55, *p* = 0.005, partial η^2^ = 0.49), but not for the x- (*p* = 0.81) or y- (*p* = 0.67) axis.

**Table 1:** Peak latency and amplitude of M100, and dipole locations

RSS				Source analysis				
Lens	Latency	Amplitude	*N*	Latency	Amplitude	Talairach coordinates		
	(ms)	(fT/cm)		(ms)	(nAm)	x	y	z
+0D	132.0 ± 16.8	63.3 ± 20.2	10	121.4 ± 12.0	15.2 ± 6.5	4.6 ± 7.1	−88.4 ± 8.0	4.1 ± 8.3
+1D	139.8 ± 18.9	47.2 ± 9.9	10	129.4 ± 12.8	11.4 ± 4.0	4.0 ± 6.7	−84.8 ± 8.8	5.6 ± 7.1
+2D	146.9 ± 24.3	42.8 ± 9.4	9	133.2 ± 14.6	8.9 ± 2.8	5.3 ± 8.6	−84.2 ± 8.9	11.4 ± 10.6
+4D	165.9 ± 29.1	30.1 ± 14.4	6	158.5 ± 18.9	8.2 ± 3.9	8.0 ± 10.9	−84.0 ± 9.2	9.4 ± 10.3

## Discussion

### Generator of M100

The field distribution in isocontour maps, polarity reversal between the upper and lower visual fields, and dipole location around the calcarine fissure indicate that the main generator of M100 was the primary visual cortex (V1), which is consistent with previous findings observed using VEP [16] and VEF [17]. Since this component of VEPs or VEFs is sensitive to the luminance and spatial frequency of the stimulus, its latency markedly varies across studies. For example, in a MEG study by Portin et al. [17], the main MEG response, which peaked at approximately 75 ms, appeared to correspond to our M100 showing almost the same response properties, except for the latency. In their study, the pattern reversal of checkerboards with white/black luminance of 60 and 1 cd/m^2^ was used, which was markedly brighter than ours (0.16 cd).

We found that V1 activity was strongly affected by refractive errors, which was congruent with the findings of previous fMRI studies showing myopia-induced reductions in BOLD signals in and around V1 [11, 12]. In these studies, the extent of the V1 activity reduction was approximately 20 % by a +8D lens [12] and 10 % by +1D lens [11], which was slightly smaller than the value obtained in the present study (25 % by +1D). This may have been due to differences in the methodology used, namely, a VEF component is more sensitive to synchrony in neuronal firing than BOLD changes. Therefore, electrophysiological measures appear to be slightly superior for detecting changes in refractive states. On the other hand, in VEP and VEF recordings, in contrast to BOLD recordings, V1 activity under the full-field stimulation was problematic because the field activities of neurons in the upper and lower banks of the calcarine fissure canceled each other out. The present results supported this by showing that the magnetic response to the full-field stimulation was approximately a sum of polarity-reversed responses to the upper and lower visual field stimulations (Fig. 1b). Therefore, the lower field stimulation may have the advantage of obtaining cortical responses with a high S/N ratio over the upper or full-field stimulations in VEP and VEF, at least when measures target V1 activity.

### Sources other than V1

The results of dipole analyses showed that a stronger refractive error resulted in a superior location of the estimated dipole. This result indicated that sources other than V1 participate in shaping M100 under strong defocusing. One probable candidate is V6 [15, 17–19], which is located superior to V1, generates an antero-superior going current, and thereby gives rise to magnetic fields similar to those from V1 at a slightly superior position. Furthermore, V6 is known to be less sensitive to spatial frequencies [15]. Therefore, the contribution of V6 to M100 relative to that of V1 is expected to increase under induced refractive error states. Another candidate is V3, which is located lateral to V1, becomes active slightly later than V1 and is also less sensitive to spatial frequencies than V1 [15]. In the present study, a subject who displayed the largest movement of the V1 dipole by inducing refractive errors showed a quadrupole pattern field distribution that was attributed to V3 activity [19] for the +4D condition. It is highly possible that V3 activity was absent or very weak at the V1 peak under the 0D condition, while V1 and V3 are simultaneously active around the later V1 peak under the +2D and +4D conditions. Therefore, when refractive errors are evaluated using VEPs or VEFs, it is important to note that temporally overlapping cortical sources may affect the results obtained and that stimulus parameters such as luminance and spatial frequency are crucial for determining the relative contribution of each cortical activity.

### Effects of refractive errors

As shown in previous VEP studies, refractive errors strongly influenced the latency and amplitude of VEFs. The present study found a significant difference between 0D and +1D for the latency and amplitude of M100, thereby supporting the view that electrophysiological measures are applicable for delicate adjustments to a lens. The difference of 8 ms between the 0D and +1D conditions was larger than the values of 2–7 ms reported in previous studies using pattern reversal (100–170 cd/m^2^) [7, 10, 13, 20]. In terms of the amplitude, the 25 % decrease from the control response for the +1D condition was similar to that reported by Anand et al. [10] at approximately 20 % using transient VEPs evoked by a checkerboard pattern with white checks of 122.9 cd/m^2^. Another study using a checkerboard pattern stimulus of higher luminance reported a decrease of 14.5 % [6]. Therefore, sensitivity to the subtle changes in refractive states in the present study was not different from or slightly superior to those obtained using brighter checkerboard pattern stimuli. From the viewpoint of subject discomfort, a simple and sharp stimulus of lower-luminance may be better than a bright and flickering stimulus.

## Conclusions

The present study showed that the VEF component, M100, evoked by a low-luminance simple grating, was sufficiently sensitive to the refractive state in order to detect subtle changes as small as 1D. Since M100 arises from V1 and the directions of the intracellular currents of M100 are opposite between neurons in the upper and lower lips of the calcarine fissure, stimulation of the lower visual field, which elicits larger responses than the upper field, appears to be suitable for assessing refractive states. In order to objectively measure refractive errors in individuals, accuracy as well as the load on a subject such as the recording time and stimulus brightness need to be considered. The present results will assist in establishing a method for the reliable measurement of refractive errors in individuals. Further studies are necessary to confirm whether the present findings are applicable to refraction errors other than myopia such as astigmatism.

## Abbreviations

MEG: magnetoencephalography
RSS: root sum square
V1: primary visual cortex
VEF: visual evoked magnetic field
VEP: visual evoked potential
